# Complaint of Testicular Discomfort in Patient with Esophageal Adenocarcinoma

**DOI:** 10.1155/2021/6692578

**Published:** 2021-04-19

**Authors:** Kevin Krughoff, Alan Schned, Bing Ren, Vernon M. Pais

**Affiliations:** ^1^Department of Surgery/Section of Urology, Dartmouth-Hitchcock Medical Center, Lebanon, NH, USA; ^2^VA Medical Center, White River Junction, VT, USA

## Abstract

We report a case of esophageal cancer with solitary metastasis to the testicle in a 71-year-old man. The tumor was picked up on physical exam following new onset complaints of pain and swelling. While most testicular masses in older men are due to lymphoma, this case highlights the need to consider metastatic disease as a source of new symptoms in patients with a recent cancer diagnosis.

## 1. Introduction

The evaluation of scrotal discomfort requires consideration of a wide variety of etiologies, and the corner stone of assessment remains the physical exam. New testicular masses in men over the age of 60 are often due to lymphoma, but in a patient with a recent cancer diagnosis and new onset of pain, the consideration for metastatic disease should remain on the differential.

## 2. Case Presentation

**Figure 1:**
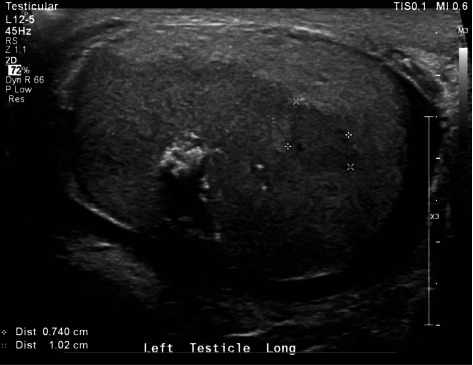
Scrotal ultrasound.

**Figure 2:**
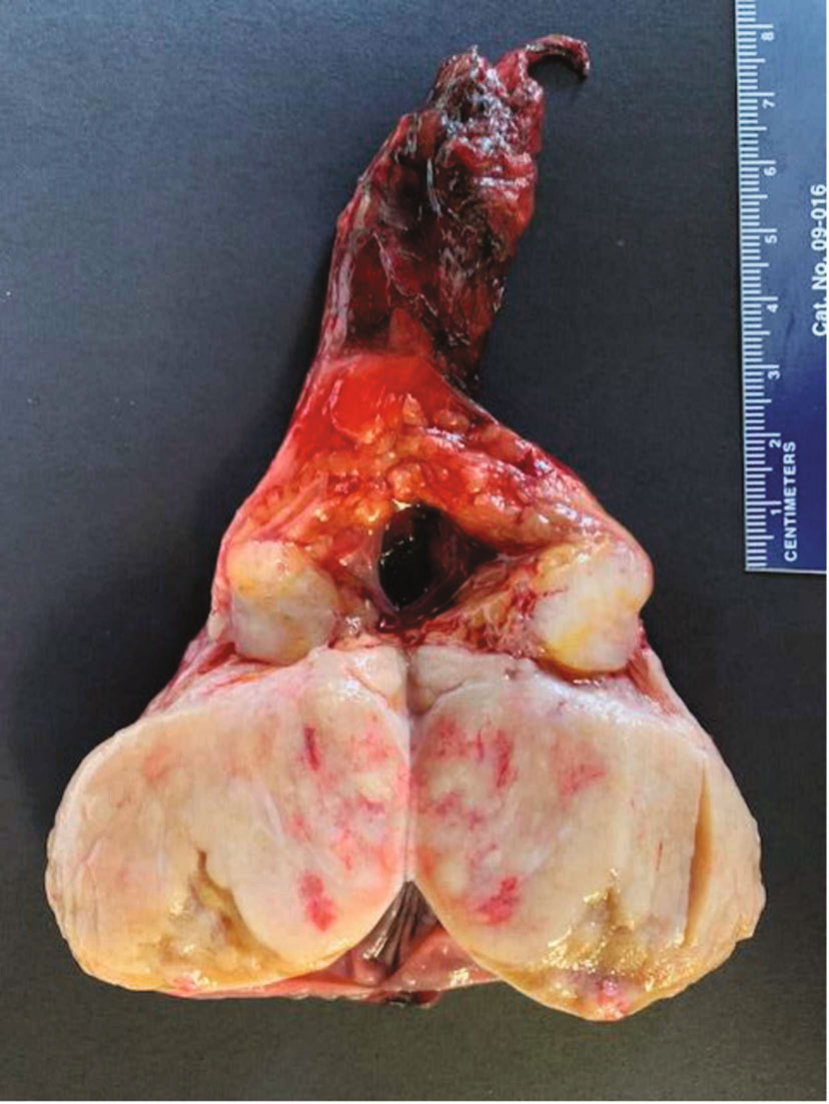
Gross specimen testicular tumor.

A 71-year-old male presented with left testicle discomfort and swelling after failing to improve on a course of antibiotics. Physical exam demonstrated a small nodule of the left testicle. An ultrasound was requested, showing a heterogeneous left testicle with small rounded hypoechoic areas, increased blood flow, and small echogenic foci along the rete testis with moderate left hydrocele (Figure 1). A left inguinal orchiectomy was performed for suspicion of testicular cancer. The orchiectomy specimen showed an enlarged testicle nearly replaced by a firm, solid, pale tan neoplasm with scattered punctate foci of hemorrhage and necrosis, 6.5 cm in maximum diameter. The tumor focally extended beyond the tunica albuginea at the hilum. A separate, well-demarcated, nearly round tumor nodule was present in the head of the epididymis (Figure 2). Tumor markers AFP (2.5), beta HCG (<1.2), and CEA (1.4) were not elevated.

**Figure 3:**
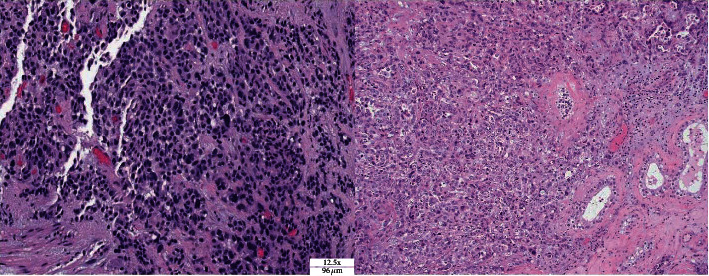
Esophageal tissue (left): sheets of neoplastic cells with high nucleus/cytoplasm ratio and nuclear hyperchromasia and pleomorphism. Immunohistochemical staining was positive for monoclonal CEA and negative for p63, synaptophysin, and chromogranin, supporting a diagnosis of poorly differentiated adenocarcinoma (H&E; ×12.5). Testicular tissue (right): poorly differentiated and loosely cohesive carcinoma with predominantly solid and some focal glandular architecture demonstrating considerable pleomorphism. Adjacent seminiferous tubule (H&E; ×10).

Immunohistochemical work-up was negative for multiple germ cell markers, including CD30, OCT3/4, PLAP, c-kit, and glypican and was positive for multiple epithelial markers, including cytokeratin AE1/3, cytokeratins 7 and 20 (focal), and epithelial membrane antigen. There was strong positivity for both monoclonal and polyclonal CEA and focal positivity for CDX-2. Markers for other potential primary tumor sites, including TTF-1 (lung), NKX 3.1 (prostate), PAX8 (renal), calretinin (mesothelial), and inhibin (adrenal plus testicular sex cord stromal tumors), were all negative. Histologically, the tumor in the testis was markedly similar to that in the esophagus, with both showing a majority solid growth pattern and focal glandular differentiation (Figure 3). Notably, both neoplasms showed positive staining patterns for CDX-2 and CEA, markers for a number of adenocarcinomas of gastrointestinal origin. Final pathology was consistent with metastatic esophageal cancer.

Ten months prior to orchiectomy, the patient had a salvage Ivor Lewis esophagectomy for esophageal adenocarcinoma, initially treated with neoadjuvant chemoradiation. Final pathology showed poorly differentiated adenocarcinoma of the esophageal gastric junction (ypT3N0) with negative margins and 0 of 22 examined lymph nodes. PET CT scans were obtained at initial diagnosis (21 months prior to orchiectomy) and preceding esophagectomy, both showing no distant metastases. A CT 6 months following esophagectomy was also unremarkable for metastatic disease. Finally, a third PET CT scan performed after orchiectomy suggested left periaortic region nodal metastasis but, again, no additional visceral metastases. Due to epididymal tumor involvement, he was then treated systemically with FOLFOX.

## 3. Discussion

Metastasis to the testicle from any cancer type is rare, with one autopsy study showing an incidence of 2.5% [1]. Metastatic testicular tumors are generally reported in older patients with a peak incidence in the fifth decade, with most originating from the prostate, lung, or colon [2–4]. Less commonly reported sites are the kidney (2), intestine (2), adrenal gland (1), malignant melanoma (1), and retinoblastoma (1) [2, 3]. In some cases, the testicular tumor discovery precedes that of the primary tumor, thus emphasizing a thorough immunohistochemical workup [5].

The lower esophagus is the most common site of esophageal cancer and the most common origin for unexpected metastases. Systemic organ metastases for esophageal cancer are as frequent as intrathoracic recurrences, with the most common sites being the regional lymph nodes, liver, lung, and bone [6]. Metastasis to the testicle with involvement of the epididymis, spermatic cord, and adjacent testicular tissues was reported once in the context of esophageal cancer with prior lymph node and cutaneous metastasis [7]. In the present report, the testicle constituted the only metastatic site despite negative surgical node status and repeated staging studies with no evidence of metastatic disease. The survival time for esophageal cancer patients decreases appreciably when recurrences are found at other locations, and the lymph nodes are uninvolved [8]. The patient went on to receive further chemotherapy following testicular diagnosis.

The esophageal origin of the testicular tumor in this case was based on morphologic similarities to the esophageal cancer, a similar immunohistochemical staining pattern, and an absence of other putative primary sites. The route of spread from a visceral cancer to the testicle in cases such as this is speculative, with previous reports suggesting lymphatic spread from the prostate, venous spread from the kidneys, or direct extension from retroperitoneal tumors [9]. The complex anatomical pathway of the esophageal lymphatic network has been found to show lymphatic nodal skip spread, both retrograde and bidirectional [2–4], which may lend support to lymphatic spread to the testicle in this case. Despite an uncommon etiology for scrotal pain, testicular metastasis should be considered on the differential when evaluating new onset scrotal discomfort in a patient with a prior cancer diagnosis.
